# Isolated adrenocorticotrophic hormone (ACTH) deficiency presenting with recurrent hypoglycemia and hyponatremia

**DOI:** 10.1097/MS9.0000000000002943

**Published:** 2024-12-24

**Authors:** Saif Ur Rehman, Marium Aisha, Aliena Badshah, Laila Hassan, Habiba Noor, Qaisar Ali Khan, Ravina Verma

**Affiliations:** a Khyber Teaching Hospital MTI KTH, Peshawar, Pakistan; b Bhatti Hospital and Maternity Home, Sukkur, Pakistan; c Lady Reading Hospital, Peshawar, Pakistan; d Hayatabad Medical Complex, Peshawar, Pakistan; e School of Medicine, St. Georges University, True Blue, Granada

**Keywords:** ACTH, case report, hyponatremia, pituitary hormones, secondary adrenal insufficiency

## Abstract

**Introduction:**

Isolated adrenocorticotropic deficiency (IAD) is a rare disorder characterized by secondary adrenal insufficiency (AI) with low or absent cortisol production, normal secretion of pituitary hormones other than ACTH, and the absence of structural pituitary defects. This case report highlights the atypical presentation of IAD, presenting with recurrent hypoglycemia and hyponatremia.

**Case presentation:**

A 20-year-old female presented to the emergency department with a loss of consciousness. Initial laboratory evaluation revealed hypoglycemia and hyponatremia. She had a history of recurrent hospitalization for the same problem for 4 months. Further investigations showed deceased cortisol and adrenocorticotrophic (ACTH) levels. The rest of the pituitary hormones were normal. The patient was diagnosed with a case of Isolated ACTH deficiency and was started on oral prednisolone, and her symptoms resolved with no recurrence of the symptoms.

**Clinical Discussion:**

The etiology of acquired IAD remains largely unclear. However, it is sometimes associated with other autoimmune diseases, such as Hashimoto’s thyroiditis. Idiopathic isolated ACTH deficiency (IIAD) is a rare condition that can present either congenitally (CIIAD) or later in life as adult idiopathic isolated ACTH deficiency (AIIAD).

**Conclusion:**

Isolated ACTH deficiency can rarely present with hyponatremia concurrent with recurrent hypoglycemic episodes and hypotension. However, further research is needed to find the pathophysiology behind it.

## Introduction

**Highlights**

- Isolated adrenocorticotropic deficiency (IAD) is a rare disorder, characterized by secondary adrenal insufficiency (AI) with low or absent cortisol production, normal secretion of pituitary hormones other than ACTH, and the absence of structural pituitary defects.
- Isolated ACTH deficiency can rarely present with hyponatremia concurrent with recurrent hypoglycemic episodes and hypotension.
- The case underscores the importance of recognizing atypical presentations of endocrine disorders, such as IAD, which may manifest with symptoms deviating from the typical clinical picture.

Isolated adrenocorticotrophic hormone (ACTH) deficiency (IAD) is a condition characterized by insufficient ACTH levels, occurring without detectable pathology in the pituitary gland or hypothalamus. This deficiency leads to adrenal dysfunction, disrupting the synthesis and release of adrenal hormones. Adrenal insufficiency can present as primary, arising from adrenal gland dysfunction, secondary, due to deficient ACTH secretion by the pituitary gland, or tertiary, resulting from impaired corticotrophin-releasing hormone secretion at the hypothalamic level^[1]^. The prevalence of isolated ACTH deficiency is estimated to be around 1 in 1 000 000 individuals, making it a rare condition. However, its true incidence may be underestimated due to its nonspecific clinical presentation and potential for misdiagnosis^[2]^. Major categories of IAD include idiopathic IAD and acquired IAD^[1]^.

The etiology of acquired IAD remains largely unclear. However, it is sometimes associated with other autoimmune diseases, such as Hashimoto’s thyroiditis^[3]^. Idiopathic isolated ACTH deficiency (IIAD) is a rare condition that can present either congenitally (CIIAD) or later in life as adult idiopathic isolated ACTH deficiency (AIIAD). AIIAD is characterized by secondary adrenal insufficiency, marked by low ACTH levels and diminished cortisol production, alongside normal secretion of pituitary hormones other than ACTH^[4]^.

IIAD specifically involves the pituitary-adrenal axis (HPA axis), with individuals exhibiting low serum cortisol and ACTH levels. Unlike other forms of adrenal insufficiency, IIAD typically does not affect the function of other hormone axes, such as the thyroid, gonads, prolactin, or growth hormone. Pituitary magnetic resonance imaging (MRI) typically appears normal or may reveal empty sella syndrome, indicating the pituitary gland’s flattened or vacant appearance^[5]^.

This case report highlights the distinctive clinical presentation of idiopathic isolated ACTH deficiency, characterized by recurrent hypoglycemia accompanied by hyponatremia. The work has been done following the Surgical Case Report (SCARE) 2023 guidelines^[6]^.

## Case presentation

A 20-year-old female patient presented to the emergency department of a tertiary care hospital with a loss of consciousness. The patient started palpitations, drowsiness, and cold sweating, followed by loss of consciousness. There was no evidence of seizures, trauma, or any acute psychological incident before the event. On arrival, the patient had a vital sign of blood pressure of 100/60 mmHg, pulse of 78 beats per minute (regular), body temperature of 98^0^F, and oxygen saturation of 96%. Initially, the random blood sugar of the patient was checked and came out to 33 mg/dl. A blood sample was withdrawn, and a 50% dextrose infusion was started. The patient responded and gradually regained her consciousness. On further inquiry, the patient also complained of epigastric pain, anorexia, nausea, vomiting, and undocumented weight loss. The patient had multiple visits to the emergency department for the same condition in the last 2 months that were responded to intravenous dextrose.

She was unmarried, her family history was unremarkable for autoimmune diseases, and she had no history of tuberculosis, diabetes, oral contraceptive pills, head trauma, steroid use, or chronic infection. The patient was admitted to the medical unit for further workup.

Clinical examination of the patient was unremarkable except for low blood pressure and pallor conjunctiva, and there were no palmer creases, no pigmentation on the skin, and no injection marks. The patient experienced undocumented weight loss with a current weight of 49 kg, height of 5.4, and body mass index (BMI) of 18.5.

Based on the history of epigastric discomfort, low blood pressure, and recurrent hypoglycemic spells, a differential diagnosis of Addison’s disease, Insulinoma, Anorexia nervosa, Bulimia nervosa, and hidden infection were suspected. Initial laboratory investigations showed low levels of hemoglobin, sodium, random blood sugar, and normal levels of white blood cells (WBCs), mean corpuscular volume (MCV), platelets, potassium, chloride, urea, creatinine, bilirubin, and alanine transaminase (ALT) as shown in Table 1. Electrocardiography (ECG) revealed no acute changes. Urine R/E shows no red blood cells and pus cells in the urine. CT brain was done previously, which was normal, and the grey and white matter was differentiated, excluding any brain injury, tumor, or abscess.

**Table 1:** Initial laboratory investigations. Gram per deciliter (g/dL), liter (L), femtoliters (fL), millimoles per liter (mmol/L), milligrams per liter (mg/L), units per liter (U/L), micrograms per deciliter (µg/dL), microliter units per milliliters (µlU/mL), milligrams per deciliter (mg/dL), milliosmoles per kilogram (mOsm/kKg)

Investigations	Results	Normal range
Hematology		
Hemoglobin (g/dl)	11.1	12-16
White blood cells (/L)	8.2	4.5–11.0 × 10^9^
Mean corpuscular volume (fL)	86	80-100
Platelets (/L)	257	150–400 × 10^9^
Blood chemistry		
Sodium (mmol/L)	123	136–144
Potassium (mmo/L)	4.1	3.7–5.1
Chloride (mmol/L)	98	96–106
Urea (mg/dL)	9	5–20
Creatinine (mg/dL)	0.8	0.6–1.1
Bilirubin (mg/dL)	0.5	0.1–1.2
Alanine transaminase (U/L)	50	7–56
Random blood sugar (mg/L)	33	110–140
Total protein (g/dL)	6.8	6.0–8.3
Albumin (g/dL)	4.1	3.4–5.4
C-reactive protein (mg/dL)	0.3	<0.3
Triglycerides (mg/dL)	98	<150
Plasma osmolality (mOsm/kg)	293	275–295
Urinalysis		
Red blood cells	Negative	Negative
Leukocytes	Negative	Negative
Endocrinology		
Serum Cortisol (morning) (µg/dL)	0.6	5–25
Fasting Insulin level (µlU/ml)	0.300	After nearly 8 hours of fasting is less than 25

**Figure 1.**
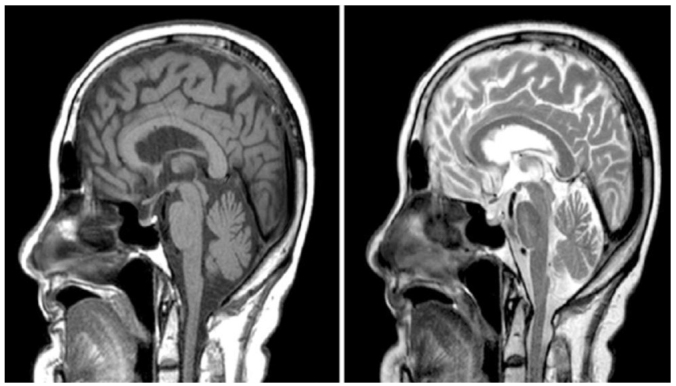
Magnetic resonance imaging (MRI) of the brain showing partial empty sella turcica.

A low level of serum cortisol was revealed, and the patient was diagnosed as a case of hypocortisolism with hyponatremia. A low fasting insulin level excluded insulinoma, which was further confirmed by the low level of c-peptide. Further investigations showed low levels of ACTH and cortisol and normal levels of plasma aldosterone and renin, suggesting central adrenal insufficiency (AI). The serum thyroid stimulating hormone (TSH), free thyroxine (FT4), and free triiodothyronine (FT3) levels were normal. Insulin-like growth factor (IGF-1), prolactin, as well as age-appropriate secretion of luteinizing hormone and follicle-stimulating hormone were also normal, as shown in Table 2. Brain magnetic resonance imaging (MRI) showed no abnormalities in the cerebral cortex, cerebellum, or brainstem, except partial empty sella turcica (Fig. 1).

**Table 2:** Endocrinological investigations

Investigations	Results	Reference range
Thyroid stimulating hormone (mlU/L)	0.54	0.400–4.049
Free thyroxine (µg/dL)	7.8	5.53–11.0
Free triiodothyronine (ng/mL)	1.4	0.97–1.69
Leutinizing hormone (IU/L)	18	5–25
Follicle stimulating hormone (IU/L)	9.6	4.5–21.5
Plasma adrenocorticotrophic hormone (pg/mL)	1.00	10 and 60
Aldosterone (ng/dL)	27	3.1–35.4
Plasma renin activity (ng/dL/hr)	1.2	0.3 ± 0.05–8.7 ± 2.7
C-peptide (ng/mL)	0.025	0.5 to 2.7
Insulin like growth factor-1 (ng/mL)	88	85–350
Prolactin (µg/L)	12	< 25

These findings indicated IAD. An infusion of normal saline was started, and the patient was strictly monitored for serum sodium and random blood sugar. She had three spells of hypoglycemia on her first day of admission, for which she received an intravenous dextrose solution and responded. The patient began corticosteroid replacement therapy with intravenous hydrocortisone. Her anorexia, nausea, and vomiting resolved, and her blood pressure was around 120/70 mmHg. The hyponatremia rapidly improved, and blood tests were performed, which showed normal levels of sodium (144 mmol/L), potassium (3.9 mmol/L), and chloride (107 mmol/L). The patient was discharged on oral prednisolone 5 mg BD and was followed up after 4 weeks at our outpatient clinic. Her condition has improved, and subsequent clinical course during corticosteroid replacement therapy for about half a year has been uneventful, with no recurrence of hyponatremia or hypoglycemic attacks.

## Discussion

Isolated ACTH deficiency is a rare disorder. It is characterized by loss of adrenocorticotropic hormone and results in adrenal insufficiency^[7]^. Adrenal insufficiency is defined as impaired synthesis and release of adrenocortical hormones. It is classified into primary, where the dysfunction is within the adrenal glands themselves. Secondary ACTH deficiency happens when the defect is in the pituitary gland secretion of ACTH. Isolated ACTH deficiency is a secondary cause of adrenal insufficiency. It is defined as low or absent cortisol production despite normal secretion of other pituitary hormones and the absence of structural defects within the pituitary gland^[8]^.

The clinical presentation of isolated ACTH deficiency is variable and nonspecific. Stacpoole et al. reported the major clinical findings in 43 cases, which included weakness, fatigue, nausea and vomiting, anemia, and hypoglycemia^[9]^. Our patient’s clinical presentation closely mirrors observations reported in previous studies, characterized initially by gastrointestinal symptoms such as anorexia, nausea, and vomiting, followed by neurological manifestations including fatigue, unconsciousness, and weight loss. This symptomatology pattern corresponds closely with observations made by Iglesias et al., who identified fatigue, anorexia, and gastrointestinal symptoms as predominant features in their study cohort^[10]^.

Laboratory assessments in our case revealed concurrent hypoglycemia with hyponatremia with no pituitary gland abnormality. These findings are consistent with laboratory findings in the studies by Iglesias and Hanon^[11]^. Diagnosing IAD based solely on clinical presentation poses challenges due to the overlap of nonspecific signs and symptoms with hypocortisolism. In cases like ours, where a patient presents with hypoglycemia, hyponatremia, and hypotension without concurrent skin darkening, the initial diagnosis often leans toward secondary adrenal deficiency. Some case reports have suggested that severe hypoglycemia and hyponatremia may accompany IAD^[12]^.

Numerous studies have delved into the clinical spectrum of patients with isolated ACTH deficiency. While limited literature has observed that individuals may present with atypical manifestations, such as flexion contractures of the legs, muscle atrophy, recurrent syncope, and concurrent hyponatremia with hypoglycemia, isolated incidents of recurrent hypoglycemia and hyponatremia in cases of IAD have been documented. However, the precise underlying mechanism of their simultaneous presence remains poorly understood. This intriguing observation underscores the complexity of IAD and highlights the imperative need for further investigation into its atypical presentations.

Confirmation of IAD relies on assessing anterior pituitary function. Typically, serum levels of ACTH and cortisol are either low or normal, while other anterior pituitary hormones remain within normal ranges^[13]^. In our patient, isolated ACTH deficiency was confirmed by the presence of low ACTH levels alongside diminished plasma cortisol levels, indicative of central adrenal insufficiency. Importantly, all other anterior pituitary hormones were within normal ranges. Additionally, the normal levels of plasma aldosterone and renin further support the diagnosis of central rather than primary adrenal dysfunction.

Moreover, the etiology of IAD remains incompletely elucidated, with potential contributing factors encompassing traumatic injury, lymphocytic hypophysitis, pituitary autoimmunity (often concomitant with other organ-specific autoimmune conditions), medication use, and radiation therapy for brain tumors^[14]^. Furthermore, IAD may be associated with primary empty sella syndrome, characterized by the descent of the subarachnoid space into the sella turcica, resulting in variable degrees of pituitary gland compression in individuals lacking pre-existing pituitary abnormalities. Notably, several studies have suggested a potential link between empty sella syndrome and the pathogenesis of isolated ACTH deficiency^[15]^. In the case under consideration, the patient lacked a history of brain tumors, radiation therapy, or traumatic injuries and did not present overt signs of hypophysitis or autoimmunity. Cranial magnetic resonance imaging (MRI) revealed partial empty sella, potentially contributing to the development of IAD.

Clinical improvement, including the normalization of blood pressure, is expected within 6 hours of treatment initiation. It’s essential to identify any underlying precipitating conditions such as trauma or infections. After 24 hours of hydrocortisone therapy, dosage reduction is considered. Ideally, the patient should transition from intravenous to oral hydrocortisone and titrate to the lowest effective dose for stability^[9]^. In our case, the patient presented with moderate hyponatremia and severe hypoglycemia, both of which significantly improved with corticosteroid replacement therapy initially with intravenous hydrocortisone and was discharged on oral prednisolone 5 mg twice daily. Complications of isolated ACTH deficiency can range from severe hypoglycemia to life-threatening situations like coma and death if hypoglycemia is not promptly recognized and managed.

Ultimately, the timely diagnosis and initiation of corticosteroid replacement therapy led to a favorable clinical outcome for our patient. Nevertheless, further research endeavors are imperative to elucidate the underlying pathophysiological mechanisms of IAD and refine diagnostic and therapeutic modalities for this rare endocrine disorder. Collaborative efforts among healthcare professionals are paramount to ensure comprehensive patient care and optimize outcomes in cases of IAD.

## Conclusion

This case report illuminates a noteworthy instance of IAD, presenting with a distinctive clinical profile characterized by recurrent hypoglycemia and hyponatremia. The case underscores the importance of recognizing atypical presentations of endocrine disorders, such as IAD, which may manifest with symptoms deviating from the typical clinical picture. By elucidating this unusual presentation, the report contributes valuable insights to medical literature, facilitating earlier detection and diagnosis of similar cases in clinical practice.

## Data Availability

The datasets supporting the conclusions of this article are included within the article.

## Research registration unique identifying number (UIN)

Researchregistry (researchregistry10603).
